# Assessment of Skimmed Milk Flocculation for Bacterial Enrichment from Water Samples, and Benchmarking of DNA Extraction and 16S rRNA Databases for Metagenomics

**DOI:** 10.3390/ijms251910817

**Published:** 2024-10-08

**Authors:** Deyan Donchev, Ivan Stoikov, Antonia Diukendjieva, Ivan N. Ivanov

**Affiliations:** 1 National Reference Laboratory for Control and Monitoring of Antimicrobial Resistance, Department of Microbiology, National Center of Infectious and Parasitic Diseases, 26 Yanko Sakazov Blvd., 1504 Sofia, Bulgaria; deyandonchev@ncipd.org (D.D.);; 2 Biocampus Sofia Association, 6 Olimpiyska Street, fl.8, 1766 Sofia, Bulgaria

**Keywords:** skimmed milk flocculation, vacuum filtration, bacterial metagenomics, benchmarking, DNA extraction, water samples, taxonomic composition, MIQ score, microbial community standard, mock

## Abstract

Water samples for bacterial microbiome studies undergo biomass concentration, DNA extraction, and taxonomic identification steps. Through benchmarking, we studied the applicability of skimmed milk flocculation (SMF) for bacterial enrichment, an adapted in-house DNA extraction protocol, and six 16S rRNA databases (16S-DBs). Surface water samples from two rivers were treated with SMF and vacuum filtration (VF) and subjected to amplicon or shotgun metagenomics. A microbial community standard underwent five DNA extraction protocols, taxonomical identification with six different 16S-DBs, and evaluation by the Measurement Integrity Quotient (MIQ) score. In SMF samples, the skimmed milk was metabolized by members of lactic acid bacteria or genera such as *Polaromonas*, *Macrococcus*, and *Agitococcus*, resulting in increased relative abundance (*p* < 0.5) up to 5.0 log fold change compared to VF, rendering SMF inapplicable for bacterial microbiome studies. The best-performing DNA extraction protocols were FastSpin Soil, the in-house method, and EurX. All 16S-DBs yielded comparable MIQ scores within each DNA extraction kit, ranging from 61–66 (ZymoBIOMICs) up to 80–82 (FastSpin). DNA extraction kits exert more bias toward the composition than 16S-DBs. This benchmarking study provided valuable information to inform future water metagenomic study designs.

## 1. Introduction

Freshwater and marine ecosystems are vital for human survival and evolution and are commonly studied using targeted or shotgun metagenomics [1,2,3]. Metagenomics studies of these environments involve extracting nucleic acids from samples to analyze the microbial communities and their functional characteristics [4,5].

Regardless of methods, study designs, and goals, samples of various water volumes are collected, transported to laboratories, and optimally processed immediately to avoid biased results [6,7]. The initial step of most bacterial metagenomics studies on water samples involves extracting biomass (also called enrichment) from the water content [8]. Most often, water samples include small particles such as soil, rocks, and plant residues to which microorganisms and/or extracellular DNA may adhere. To capture all microbial content, these particles are ideally retained in the final sample [9,10], including those present as planktonic forms. For this purpose, several techniques have been developed and used to varying degrees, namely, vacuum filtration (VF), ultracentrifugation (UC), skimmed milk flocculation (SMF), and polyethylene glycol (PEG) precipitation. UC and PEG precipitation are applied almost exclusively for viral and phage metagenomics [11,12,13].

VF is mainly used for the characterization of bacterial and fungal communities and is by far considered the gold standard [14]. It is a straightforward size exclusion or inclusion method used in metagenomics to separate microbial cells and sample debris from the water content by vacuum as a pressure-driven factor. However, there are numerous factors that influence the filterability during vacuum filtration, such as membrane protein-binding affinity, surface charge, hydrophobicity, pore size and structure, and roughness [15]. Additionally, the size, shape, flexibility, charge, and hydrophobicity of cells also influence the potential of bacteria to flow through the filter, and lastly, the particulate nature of the sample suspension itself [15]. For these reasons, research on filtration methods is still evolving [15] and alternative approaches are being sought.

The SMF might be considered a viable option for bacterial enrichment. It was originally developed to concentrate viruses from coastal waters [16], later widely applied to SARS-CoV-2 [17], as well as others [18,19,20,21]. By design, it is straightforward, affordable, and quick to perform, and requires simple laboratory equipment. The flocculation is achieved at 3.5–4.0 pH, where casein proteins (net positive charge) interact with viral particles from water samples, which carry a net negative charge due to their functional surface groups, such as carboxylates and phosphates [21]. The reaction solution is usually agitated at low rpm for approx. 2 h, during which, through electrostatic interactions, flocs (virus-protein complexes or aggregates) are formed and settle out of the solution either naturally or facilitated through centrifugation at 3500× *g* for 30 min [22]. Previously, SMF has been used for simultaneous concentration and quantification of waterborne viruses, bacteria, and protozoa [23] mainly for water control purposes and microbial risk assessment studies. However, there is a literature gap on its applicability in bacterial microbiome studies. Therefore, we used both 16S rRNA and shotgun metagenomics data from water samples to assess the applicability of SMF for bacterial metagenomics.

Another critical step is the extraction of DNA from filters or precipitates. Numerous studies have assessed the impact of various DNA extraction protocols on the quantitative analysis of bacterial biomass [24,25,26,27,28,29], highlighting the need for a standardized protocol that yields reproducible results to facilitate cross-study comparisons, especially in research areas with future health diagnostic perspectives such as the profiling of the human gut microbiota. For instance, according to the largest current comparative study based on shotgun sequencing evaluating the bacterial extraction performance of 21 fecal DNA extraction protocols [24], Protocol Q, which is a slightly modified version of Qiagen’s QIAamp DNA Stool Mini Kit, has been proposed as the standard protocol providing the best results for bacterial DNA extraction from human feces. To date, there is no equivalent DNA extraction protocol for aqueous environmental samples [29].

Next, in the case of targeted bacterial metagenomics, after sequencing and initial quality control of sequencing data, 16S rRNA databases (16S-DBs) are utilized to infer the bacterial taxonomic composition. While the de novo clustering approach is often the choice for initial observation as the composition is not influenced by 16S-DBs, it is not optimal for cross-study comparisons [30]. On the other hand, closed or open-reference clustering approaches are still widely preferred [31], although they utilize 16S-DBs for clustering, which introduces bias. Several studies benchmarked their performance previously [28,32,33,34], but they undergo constant updates that prompt additional evaluation.

To address the last two problems, we optimized an in-house protocol that incorporates elements from other published protocols and applied it to a 10-species microbial community standard (MCS) alongside four other commercial DNA extraction kits. The MCS samples were then subjected to 16S rRNA metagenomics, and the resulting sequence data were used to benchmark the DNA extraction kits and six well-established as well as more recently published 16S-DBs. For effective comparison, we used the measurement integrity quotient (MIQ) score, which quantifies the difference between the observed and the expected composition [35]. This study covers three critical aspects of metagenomics workflows: the applicability of SMF for bacterial metagenomics on real samples, the evaluation of our in-house DNA extraction method, and the comparison of 16S-DBs for taxonomic assignments.

## 2. Results

First, we evaluated the bias introduced by different DNA extraction protocols and the 16S-DBs on the 16S metagenomics MCS datasets (*n* = 8). In total, we scored factors such as DNA yield, A_260/280nm_, A_260/230nm_, species-level taxon accuracy rate (TAR), genus-level taxon detection rate (TDR), MIQ, and the percent of reads that mapped to the reference sequences, failed quality filter, failed to merge, or were chimeric (Supplementary Table S1). The in-house method yielded the highest amount of DNA, while the highest purity was achieved with the EurX and EZNA kits. Based on the overall results, the EurX kit was used for the shotgun studies and the evaluation of SMF.

### 2.1. Variability of MIQ Score in DNA Extraction Protocols

**Figure 1:**
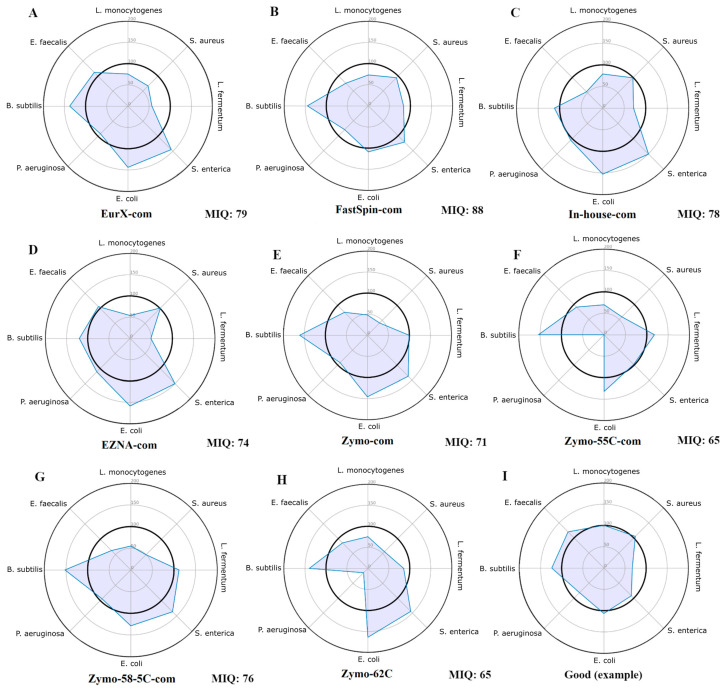
Radar plots of all MCS samples (**A**–**H**) assessed using the original Zymo 16S MIQ Calculator. These plots display the observed proportion of each organism relative to its expected value. Ideally, all points should be positioned around the 100% mark (the inner bolded circle), indicating that the observed proportions are at or close to the expected values, as shown in plot (**I**) (a non-biased MCS with a good score). The total bias in each sample was calculated as an MIQ score (higher is better), with scores > 90 considered excellent and 80–89 rated as good.

The miqScore16SPublic tool (2.6.) provided by Zymo was used to establish the bias introduced by DNA extraction kits. It was designed to generate amplicon sequence variants (ASVs) instead of operational taxonomic units (OTUs) and calculate an MIQ score for each MCS, assigning a value between 0 (indicating bias) and 100 (indicating no bias) based on the comparison of observed versus expected composition. Complete reports are available in Supplementary File S1 and only the radar plots were provided in Figure 1. Results indicate that the samples isolated by the FastSpin Soil kit showed the least biased score (88 MIQ), followed by EurX and the in-house method, whereas the worst MIQ scores were in the Zymo kit and its datasets with varying annealing temperatures.

### 2.2. Bias Introduced by 16S rRNA DBs and/or DNA Extraction Kits on MCS Samples

**Figure 2:**
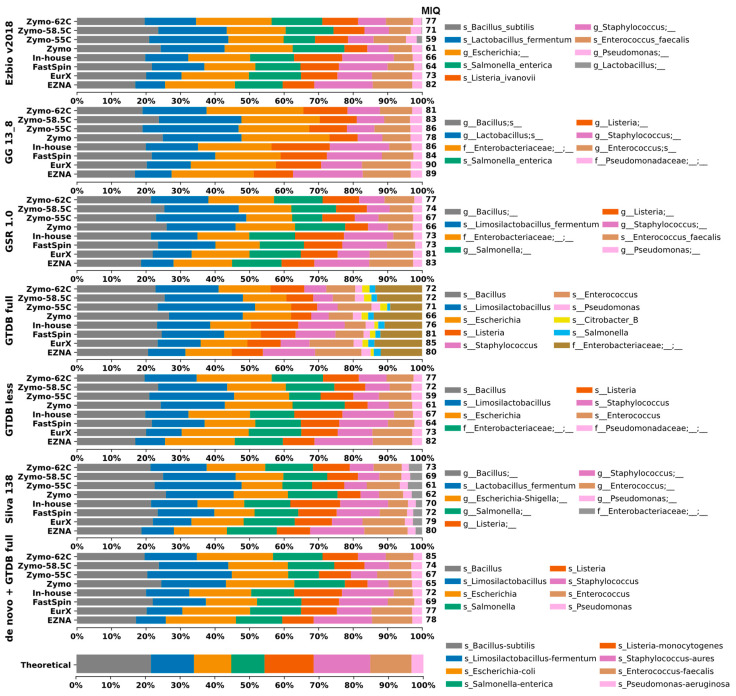
Taxa bar plots of the eight MCS samples analyzed with closed-reference clustering with each 16S-DB separately and de novo clustered datasets identified with GTDB-full. A database-specific legend with taxa identification (lowest rank) is provided on the right side. For easier interpretation, the resulting MIQ scores are provided right next to the bars. A higher MIQ score represents taxonomic composition closer to the expected one (bottom).

Herein, we assessed to what extent the 16S-DBs might introduce bias in taxonomic composition. Closed-reference OTU clustering at 99% was applied to the MCS samples. Unfortunately, the tool miqScore16SPublic is incompatible with taxonomic tables generated from external sources. As a workaround, we implemented its intrinsic MIQ score formula in a simple Python script, allowing the calculation to be applied to taxonomic tables regardless of their origin. Taxonomic composition plots of all MCS samples are visualized in Figure 2.

**Figure 3:**
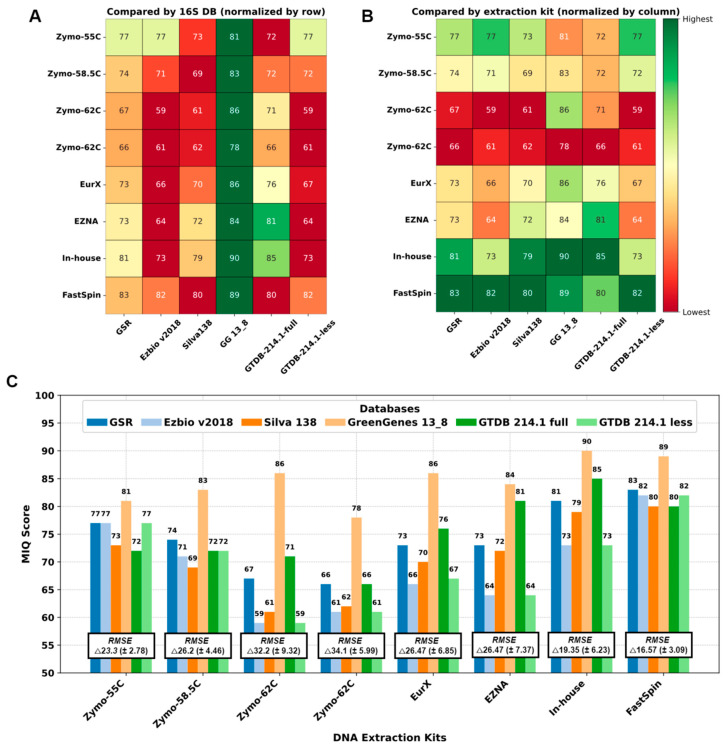
Performance of DNA extraction kits and 16S-DBs by MIQ score: (**A**) Comparison of 16S-DB. (**B**) Comparison of DNA extraction kits/annealing temperatures. Green is better and red is worse. (**C**) Bar graph with MIQ scores and average root mean square error (RMSE) values.

The GTDB-full and Silva DBs generated more OTUs compared to the remaining 16S-DBs. This could be considered both beneficial and negative depending on the study’s purpose. However, in this case, the additional OTU Citrobacter_B in GTDB-full that should not be present in the MCS sample skewed the results, resulting in lower MIQ scores (Figure 2 and Figure 3).

We compared the 16S-DBs and DNA extraction protocols using MIQ scores in parallel, and the results are shown in Figure 3. First, 16S-DBs were compared (Figure 3A), and the best compositions with the least bias were yielded by GG_13.8, followed by GSR and GTDB-full. Interestingly, the GG_13.8 DB failed to differentiate between *Escherichia coli* and *Salmonella enterica*, combining them into a single family-level OTU group (*Enterobacteriaceae*). Unfortunately, there was no adequate approach to separate this 99% clustered OTU group for the MIQ calculator to precisely quantify the bias for each microorganism. Therefore, for GG_13.8 specifically, we treated both *E. coli* and *S. enterica* as a single organism and adjusted the reference expected composition in the MIQ score script. As a result, all the MIQ scores from GG_13.8 are elevated compared to the remaining 16S-DBs and should be interpreted with caution.

Next, we compared the performance of different DNA extraction kits (Figure 3B), regardless of the 16S-DBs used. Except for the ZymoBIOMICs with a primer annealing temperature of 55 °C, all the Zymo variants performed worse compared to the other kits. The FastSpin kit produced the highest MIQ scores (82.6 on average) with all 16S-DBs. The second-best results were scored by our in-house protocol.

Interestingly, all the 16S-DBs aside from GG_13.8 yielded comparable results across the DNA extraction kits, with scores ranging from 59 to 71 in the worst-performing sample (Zymo-62C) and 80 to 83 in the best sample (FastSpin). These results suggest that the choice of DNA extraction kits had a greater impact on the final MCS composition. The bias observed in MCS samples could not be compensated by using a better-performing 16S-DB (as seen with both repetitions of Zymo-62C in Figure 3C). Conversely, a sample treated with a good-performing DNA extraction kit yielded a taxonomic composition resembling the expected outcome, regardless of the 16S-DB used.

### 2.3. Taxa Identification Efficiency of 16S rRNA DBs on De Novo Clustered MCS Samples

While taxonomic composition is the first criterion to assess the bias in MCS samples, TAR and TDR are two important factors that essentially evaluate how 16S-DBs perform the taxonomical identification. Although we presented the results as values for each 16S-DB, one should note that they are heavily influenced by the amplified region, and the choice of the OTUs vs. ASVs approach, and should not be considered entirely as drawbacks to the 16S-DBs.

The results of TAR and TDR were calculated with data from 99% de novo clustered OTUs as the taxonomic composition of all resulting OTUs is the same regardless of the DB, which provides an equal basis for comparison. The 99% OTU-generated results tend to underrepresent *Enterococcus faecalis* by splitting it into two separate OTUs *g_Enterococcus* and *s_Enterococcus faecalis* with similar relative frequency values in all extraction kits, thus skewing the total microbial composition. For a small MCS with only eight bacterial strains, results did not vary drastically, and also the TAR and TDR scores at each taxonomic level were identical. At the species level, accurate identification varied from 1/8 bacteria for Silva, 2/8 for GSR, and up to 4/8 for Ezbio, while for the genus level, it was between 6/8 and 8/8 (Figure 2). GG_13.8 and GTDB were designed for genus-level identification; therefore, species-level resolution was not possible and not discussed. Only one case of misclassification was recorded, namely, *Listeria monocytogenes* identified as *Listeria ivanovii* by Ezbio. Underclassifications by two taxonomic levels were observed for *Salmonella enterica* by GTDB-full, GTDB-less, and Silva and for *Escherichia coli* by GG_13.8 and GSR. Additionally, *P. aeruginosa* was underclassified by two ranks by GG_13.8 (Figure 2).

### 2.4. Evaluation of Skimmed Milk Flocculation

**Figure 4:**
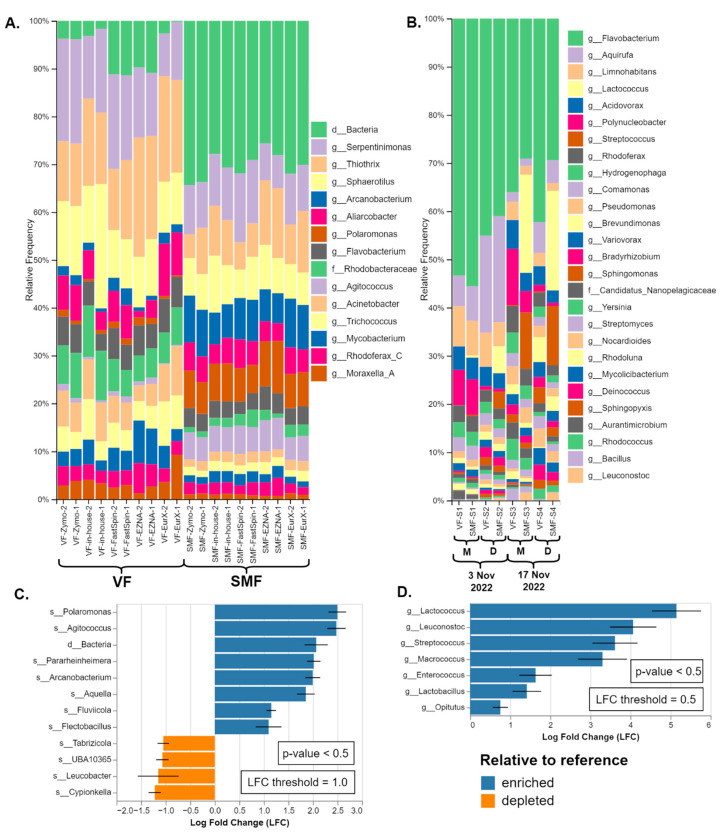
Comparison of the taxonomic composition of SMF and VF: (**A**) Duplicate datasets of the amplicon metagenomics samples from River Perlovska per DNA Extraction kit. (**B**) Datasets of shotgun metagenomics samples from River Iskar. The sample pairs (VF and SMF-treated) were ordered by sampling date and location (M—villa area Mechkata, D—Dragushinovo village). Differential abundance analysis of (**C**) amplicon and (**D**) shotgun samples with all VF samples pooled as a reference compared to the SMF samples. Only taxa with *p*-value < 0.5 and with log fold change (LFC) ≥ 1.0 (**C**) and 0.5 (**D**) are presented.

SMF and VF were compared only on real samples as the MCS by Zymo is not designed to be pre-treated before DNA extraction as cells are stored in DNA/RNA Shield^TM^ and are partially lysed. The relative taxonomic composition of both types of datasets was presented in Figure 4A,B. In all sample pairs (a pair being VF and SMF-treated), noticeable separation of SMF- and VF-treated samples was observed in the PCoA plots (PERMANOVA: F = 26.6, R^2^ = 0.6, *p* < 0.001), as shown in Supplementary Figure S1 While the significant separation based on the Bray–Curtis beta diversity index indicated that the taxonomic composition of VF and SMF-treated samples differed, differential abundance analysis (DAA) was employed to identify which taxa were the driving factors of this effect. The results are available in Figure 4C,D and the full-length plots in Supplementary Figures S2 and S3.

In the 16S rRNA amplicon datasets (River Perlovska), the genera *Polaromonas* and *Agitococcus* and an OTU identified at high taxonomic rank were observed to be the highest overrepresented in the SMF-treated samples. The genus *Polaromonas* was identified by GG_13.8 and the GSR at the species level as *P. naphtalenivorans*. The third OTU was either domain *Bacteria*, order *Bacteroidales*, or identified as family *Saprospiraceae* by EzbioCloud (both the free DB v2018 and their website non-free latest DB v2023.08.23). All three taxa and a few more were also shown to be significantly enriched (*p* < 0.5) with log fold change (LFC) of 2.0 or greater in all DNA extraction kits, as shown by the DAA in Figure 4C.

A similar trend was observed in the shotgun metagenomics datasets (River Iskar) but with different profiles of enriched taxa. Not all enriched taxa were visible on the bars except for the genera *Streptococcus* (brown) and *Lactococcus* (pale yellow) in SMF-S2, SMF-S3, and SMF-S4 in Figure 4B. Sankey plots are provided for better visualization of all taxa in Supplementary Figure S4. According to the DAA, the lactic acid bacteria members *Lactococcus*, *Leuconostoc*, *Streptococcus*, *Enterococcus*, and *Lactobacillus* were significantly enriched (*p* < 0.5) with LFC between 1.75 and 5.0 in the SMF-treated samples. Lastly, the genus *Macrococcus* was also significantly overrepresented and Bracken species-level hits were mostly *Macrococcus caseolyticus.* Interestingly, the genus *Streptococcus* was also detected in the amplicon metagenomics samples but was not overrepresented.

While the profiles of these enriched taxa varied across different samples and methodologies, the enrichment effect was clear and significantly influenced the final taxonomic composition. This consistency was observed despite variations in DNA extraction kits used for 16S and different sampling dates in shotgun metagenomics. Therefore, this effect appeared independently of methodology or sample type. The complete nonfiltered taxonomy tables are available as Supplementary Tables S2–S4.

## 3. Discussion

This is the first study to evaluate the applicability of SMF in bacterial metagenomics. Despite the small scale of the study design, with the use of DAA analysis, it was clearly shown that SMF skewed the taxonomic composition of real water samples, therefore rendering this SMF protocol inapplicable for bacterial enrichment in metagenomics. Interestingly, not all taxa were altered, rather only specific ones. On the contrary, a previous study on the concentration of specific species such as *Escherichia coli* and *Helicobacter pylori* SMF concluded that it could be used for the qualitative detection of those pathogens [23]. Although they proved that both species could be effectively recovered from water samples by using SMF, it remains unknown if their actual concentration was affected, as observed for other species in this study.

Skimmed milk primarily consists of protein (casein and whey) and lactose, in addition to other nutrients and minerals that could act as growth factors for bacteria, and is commonly supplied in culture media. In this regard, the genus *Polaromonas* has previously been shown to be enriched in dairy products removal tanks [36], while other studies have identified it as the third most abundant genus in mixed-species dairy biofilm within biofilters [37]. The genus *Agitococcus* was also significantly enriched in SMF-treated samples and, while no species-level identification was achieved, *Agitococcus lubricus*, a species first described in 1981, tested positive for skimmed milk proteolysis [38]. It is likely that other members of the genus *Agitococcus* would also be capable of proteolysis. Unfortunately, the most abundant significantly enriched OTU group in the sample was identified at a high taxonomic level with all 16S-DBs. This level of identification is too general and possibly unreliable, making it difficult to draw any meaningful conclusions about its potential role in SMF utilization.

Similarly, in the shotgun datasets, all the enriched lactic acid bacteria are generally found in decomposing plants and milk products, which produce lactic acid as the main metabolic end product of carbohydrate fermentation by utilizing the lactose from the skimmed milk. The acidification of the samples (pH = 3.5) during SMF, which facilitates the flocculation process, might be advantageous to their replication. Lastly, according to Bracken’s reports, most of the *Macrococcus* read hits were *Macrococcus caseolyticus*, which has again been shown to efficiently hydrolyze casein and is a natural component of the secondary microflora in cheeses and sausages [39,40]. The skimmed milk was highly likely metabolized during the 2 h incubation protocol resulting in the replication of specific taxa.

DNA extraction is a critical step in a metagenomics workflow and is known to be influenced by numerous parameters, which are challenging to evaluate comprehensively. The choice of the DNA extraction method strongly affects the detection and composition of bacterial communities [41,42]. In-house protocols and commercial products are constantly being developed and widely used, making cross-study comparisons difficult. As a result, either updated benchmarking studies or standardization efforts are required. While we developed a well-performing DNA extraction protocol, further improvements are needed to match the performance of the EurX kit (with ASVs) or the FastSpin (with OTUs). However, our in-house method could be a viable option for cost-effective research or where other protocols are unavailable. Surprisingly, the EZNA Universal Pathogen kit is not designed for metagenomics and is certainly not optimized to extract DNA equally from Gram-positive and Gram-negative bacteria, as the cell wall of Gram-positive bacteria contains a thick layer of peptidoglycan. Despite this, it performed similarly to other kits, such as ZymoBIOMICs and EurX, without a bead-beating step, which is currently widely adopted and recommended to facilitate balanced lysis [42].

Next, our optimized 16S rRNA amplicon library sequencing protocol yielded good results, producing MIQ scores > 80 with most DNA extraction kits including the in-house method, which classifies them as good. Since MCS is used as a control for DNA extraction, running it in parallel with real samples helps confirm that there is minimal or no bias in the extraction process. However, benchmarking sequencing datasets from simultaneous 16S amplicon library generating protocols or commercial kits is required to fully evaluate the applicability of the 16S protocol.

In regard to the 16S-DBs comparison, we aimed to present the most sample- and primer pair-specific taxonomic identification by first truncating the reference sequences to the primer regions and then building a classifier. By doing so, the detection and identification accuracy of each 16S-DB were specifically adjusted to the primer pair used, allowing for a standardized comparison. The TAR and TDR values were not as informative as initially perceived, mainly due to the small number of bacteria included in the MCS. The results of the 16S-DB comparison presented here should be interpreted alongside the amplified region, as identification is also heavily influenced by this factor. The TAR of the resulting OTUs, clustered at a threshold equal to or below 99%, usually suffered from identification bias [43]. As anticipated, no eight out of eight TAR was achieved with the OTUs. While the most reliable identification is typically achieved with ASVs, a large portion of studies still rely on OTU clustering [32,44].

A few limitations of the study can be listed. The SMF protocol applied in this study was originally optimized for virus concentration. In the literature, SMF protocols adopted or adjusted for bacteria are lacking and additional pre-treatment steps could be implemented to inhibit bacteria growth. Ideally, MCS with a higher number of bacteria (20+) would provide more insightful results compared to the eight-bacteria MCS used here.

## 4. Materials and Methods

### 4.1. Samples

Two different sets of water samples were collected and processed separately. For shotgun metagenomic sequencing, four composite water samples (1 L each) were collected in pairs along the River Iskar from the two locations (42.367698, 23.555463—Dragushinovo village and 42.431095, 23.531900—villa area “Mechkata”) with an automatic sampler (Bühler 2000 Portable automatic water sampler, Hach UK, Manchester, UK) for a 24 h period to avoid day/night fluctuations bias on 3 November 2022 and 17 November 2022. They were transferred to the laboratory within 6 h and immediately processed. For 16S rRNA amplicon sequencing, one non-composite water sample (1 L) was collected in a sterile HDPE plastic container from a small urban River Perlovska at location 42.692164, 23.343892 and transported within 30 min to the laboratory.

**Figure 5:**
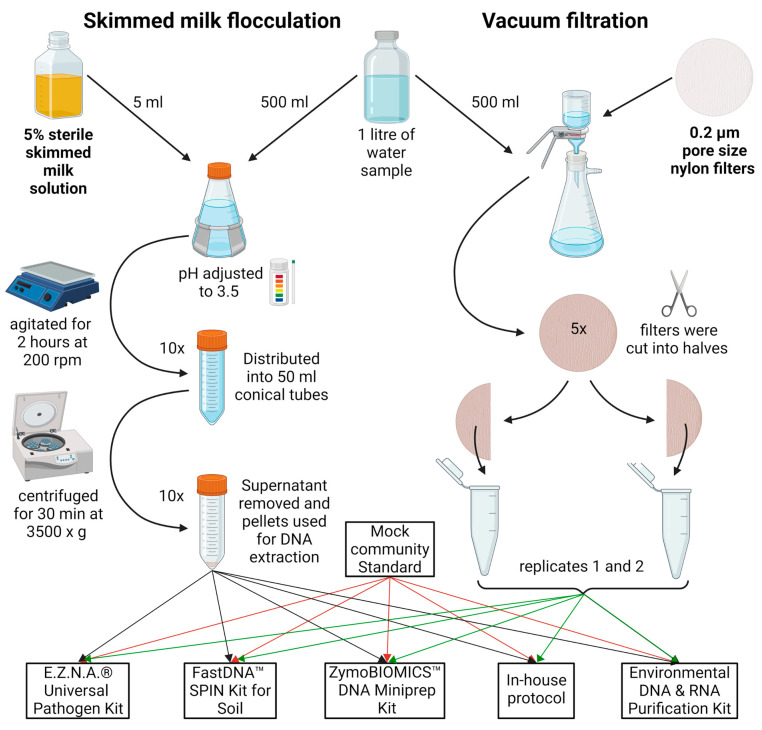
Graphical representation of the SMF and VF water treatment steps. In brief, the sample was divided into equal parts. For SMF, the sample was adjusted to 0.05% skimmed milk, with pH 3.5–4.0, agitated at slow speed for 2 h, aliquoted, and centrifuged at 3500× *g* for 30 min. For VF, the sample was further divided into 100 mL portions and filtered through 0.2 µm nylon filters, aseptically cut into smaller pieces, and added to DNA extraction tubes. A pair of two tubes with pellets/filters were resuspended in lysis buffer from each kit/protocol. The figure was created with BioRender.

All samples were divided into two equal parts of 500 mL for SMF and VF treatment. The portions from the Perlovska River were further split into five sub-portions, each subjected to a different DNA extraction method, resulting in a total of ten DNA samples (Figure 5). The MCS used here was (cat. D6300, Zymo Research, Irvine, CA, USA). The MCS mimics a mixed microbial community of 10 members (8 bacteria and 2 fungi) of a well-defined composition.

### 4.2. Skimmed Milk Flocculation

A previously described SMF protocol was used [22]. In brief, 5% skimmed milk (HiMedia Laboratories, Mumbai, Maharashtra, India) was autoclaved for 15 min at 115 °C, 18 psi. Then, 5 mL of the 5% preflocculated skimmed milk solution was added to the 500 mL sample to achieve 0.05% final skimmed milk concentration. The sample pH was adjusted to 3.5–4.0 with 1M HCl, placed on a horizontal shaker, and agitated at 200 rpm for 2 h at room temperature. It was then distributed into 50 mL conical tubes and centrifuged at 3500× *g* for 30 min at 4 °C. The supernatant was decanted and the tubes were left upside down to drain residual water for 5 min. Pellets were used for DNA extraction (see Section 2.4). 

### 4.3. Vacuum Filtration

VF was conducted with 47 mm diameter 0.2 µm pore size nylon filters (Cytiva, Marlborough, MA, USA), using Lafil 400-LF 30 Filtration System (Rocker, Kaohsiung City, Taiwan). The sample of River Perlovska was further divided among five filters as shown in Figure 5, while the entire volume of 500 mL from River Iskar samples was filtered through one filter. Regardless of the sample sets, each filter was cut sterilely into two equal halves to be further extracted in pairs. Each half was cut into smaller pieces for better homogenization and directly added to extraction tubes (see Section 2.4).

### 4.4. DNA Extraction

DNA was extracted in duplicates from Perlovska River (16S metanogemics) and MCS samples with the following kits by adhering to the manufacturer’s instructions: (1) E.Z.N.A. Universal Pathogen Kit (OMEGA Bio-Tek, Inc., Norcross, GA, USA); (2) ZymoBIOMICS DNA Miniprep Kit (Zymo Research, Irvine, CA, USA); (3) FastDNA Spin Kit for Soil (MP Biomedicals, Santa Ana, CA, USA); (4) Environmental DNA & RNA Purification Kit (EURx Sp. z o.o., Gdańsk, Poland); and (5) the in-house protocol. The in-house protocol was developed by combining elements from other published protocols [45,46] with slight modifications and optimization performed locally. The complete detailed protocol and required reagents are provided in Supplementary Table S5. DNA from the Iskar River samples was extracted with the Environmental DNA & RNA Purification Kit (EURx Sp. z o.o., Gdańsk, Poland).

### 4.5. 16S rRNA and “Shotgun” Metagenomics

The 16S rRNA V3-V4 region was amplified with previously published primer pairs Pro341F and Pro805R [46] and with an optimized 16S rRNA amplification protocol described in Supplementary Tables S6–S9. Sequencing was carried out on Illumina MiSeq V3 (2 × 300 bp). Additionally, for the Zymo miniprep DNA kit, we used the same DNA sample in three additional amplification reactions with primer annealing temperature gradient (55 °C, 58.5 °C, 62 °C).

Shotgun sequencing libraries were constructed with Illumina DNA Prep kit (Illumina, Inc., San Diego, CA, USA) with 50 ng genomic DNA input. The libraries were pooled and sequenced on NextSeq 550 with the V2.5 mid-output kit (2 × 150 bp) (Illumina, San Diego, CA, USA).

### 4.6. Bioinformatic Analysis

For comparison of DNA extraction kits and 16S-DBs on MCS samples, we used the Qiime2 platform [47] and miqScore16SPublic tool (https://github.com/Zymo-Research/miqScore16SPublic, accessed on 22 July 2024) to determine which DNA extraction kit yields microbial composition closest to the expected. Raw reads were automatically demultiplexed and trimmed from adapters in BaseSpace. The cleaned reads were submitted to the miqScore16SPublic tool to calculate MIQ scores, and the results were considered as references. Next, cutadapt v4.6 was used to remove 16S rRNA primers and remove low-quality bases (<20 Q at the 5′end, and <15 Q at the 3′end). We used six 16S-DBs and trained classifiers (*n* = 6) locally. First, full-length 16S-DBs: Silva 99%-OTU (Silva) [48], GSR-99%-OTU (GSR) [34], GTDB-214.1-99%-OTU-less (GTDB-less) [49], GTDB-214.1-99%-OTU-full (GTDB-full) [49], GreenGenes-13_8-99%-OTU (GG_13.8) [50], and EzBio-Cloud-v.2018 [51] were downloaded. Reference sequences were extracted based on in silico PCR with the 16S primer pairs, and used as input for the Naive Bayes algorithm [52] within sk-learn Python package v1.5.2 to train personalized classifiers with default parameters, which were later used for taxonomic assignments with a confidence index of 0.7 (default). Both de novo and closed-reference clustered OTUs (for each 16S rRNA DB) were obtained at 99%. De novo clustered OTUs were processed with the “evaluate-composition” plugin [53] in Qiime2 to compare TAR and TDR. Closed-reference OTUs were scored with a custom Python script (https://github.com/maddne/MIQ-calc-from-OTU-tables (accessed on 4 October 2024)), which uses the intrinsic formula for MIQ score calculation by the tool miqScore16SPublic.

Next, for a comparison of SMF and VF, we used DAA. The taxonomic assignment to raw reads was performed with Kraken2 v2.1.2 [54] with the PlusPF DB (standard plus Refeq protozoa and fungi), built in January 2023, followed by Bracken v2.8 [55] as described in protocol [56]. The resulting OTU count tables were imported into the Qiime2 platform, filtered from low-count taxa (*n* = 20 in at least 3 samples), and used for differential abundance analysis with the ANCOM-BC2 plugin [57].

## 5. Conclusions

In this study, we systematically evaluated the effectiveness of SMF for bacterial metagenomics, introduced and benchmarked an adopted in-house DNA extraction method against four commercial kits, and assessed the performance of six 16S-DBs for taxonomic identification.

The findings reveal that skimmed milk flocculation is not suitable for bacterial microbiome studies as it significantly alters the microbial composition due to the proliferation of lactic acid or casein utilizing bacteria, leading to an increased relative abundance compared to the traditional vacuum filtration method. Our in-house DNA extraction protocol demonstrated competitive performance, particularly in comparison to the commercial kits, which were optimized for minimal bias. This in-house protocol provides a cost-effective alternative for researchers with limited access to commercial kits, offering reliable results for metagenomic studies.

Lastly, the evaluation of 16S-DBs showed that while there are variances in taxonomic assignments, the choice of DNA extraction protocol has a more pronounced impact on the microbial composition than the choice of the 16S-DB. This underscores the importance of selecting an appropriate DNA extraction method to minimize biases in metagenomic studies. This comprehensive benchmarking study offers insights for the design of future water metagenomic studies, emphasizing the importance of method selection at various stages to ensure accurate and reliable microbial community profiling.

## Data Availability

All used data are included in the main text and the Supplementary Materials. Shotgun metagenomic data are available under the Bioproject PRJNA1071831. Amplicon metagenomic data are available under the Bioproject PRJNA1138176. Generated information and/or datasets analyzed during the current study are available from the corresponding author on reasonable request.

## Supplementary Materials

The following supporting information can be downloaded at: https://www.mdpi.com/article/10.3390/ijms251910817/s1.
