# Supplementary material for: Assessment of Skimmed Milk Flocculation for Bacterial Enrichment from Water Samples, and Benchmarking of DNA Extraction and 16S rRNA Databases for Metagenomics
Source: Int J Mol Sci. 2024 Oct 8;25(19):10817. doi: 10.3390/ijms251910817 (PMC11477342; doi:10.3390/ijms251910817)
Supplement: Supplementary file 1 [file ijms-25-10817-s001.zip › Suppl. Figures S1-S4.drawio.pdf]

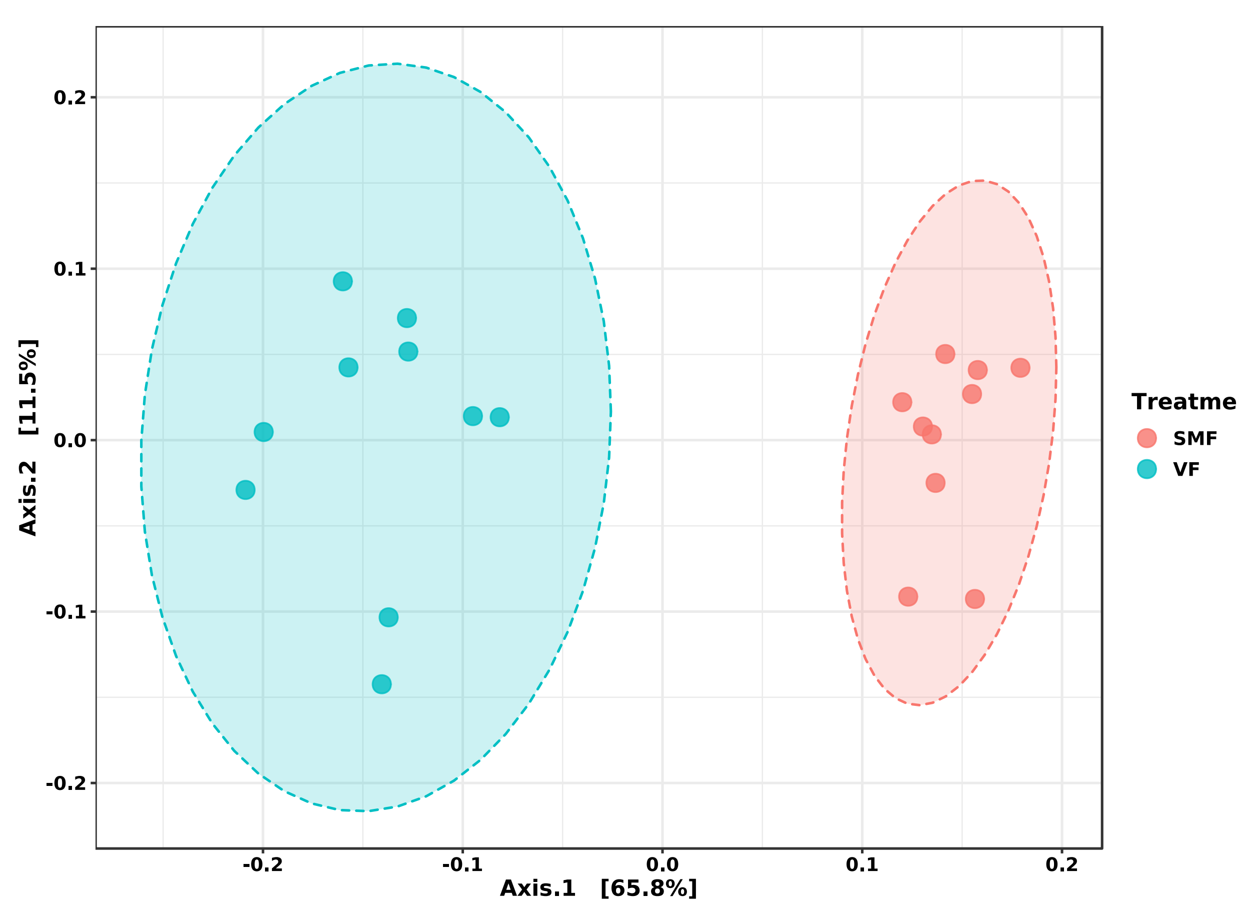

Figure S1. PCoA plots displaying the separation of the SMF-treated and VF-treated samples of 16S amplicon datasets from River Perlovska. The graph was prepared with ResistoXplorer by filtering out low count taxa (<5) with prevalence in at least 4 samples and applying total sum scaling (TSS). There is a statistically significant difference between both groups [PERMANOVA] F-value: 26.655; R-squared: 0.59691; P-value < 0.001.

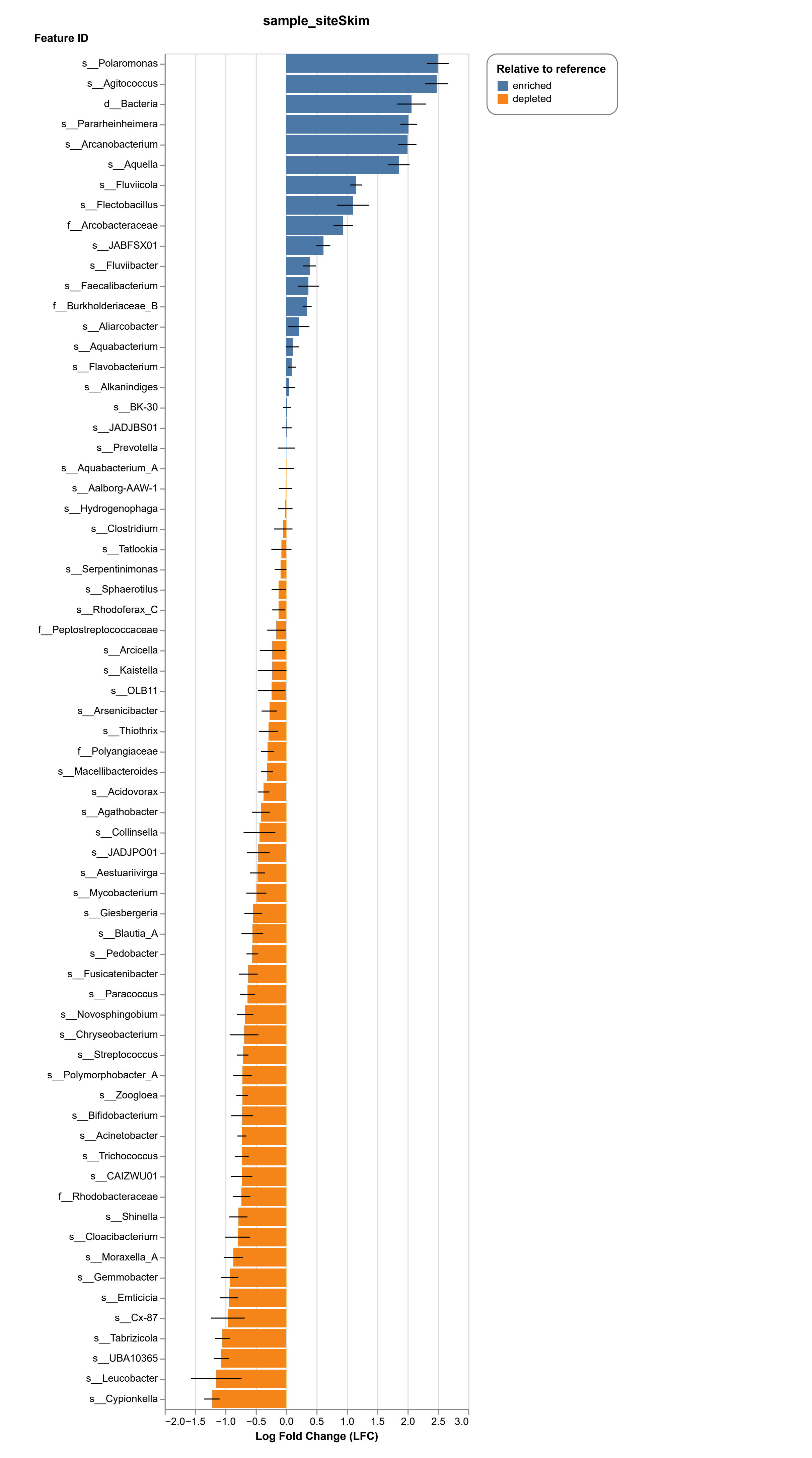

Figure S2. Differential abundance analysis in full length of the 16S amplicon shotgun metagenomics samples from Perlovska River - all DNA extraction kits were pooled in VF (reference) and SMF groups and compared. Only taxa with *p* value < 0.5 were displayed.

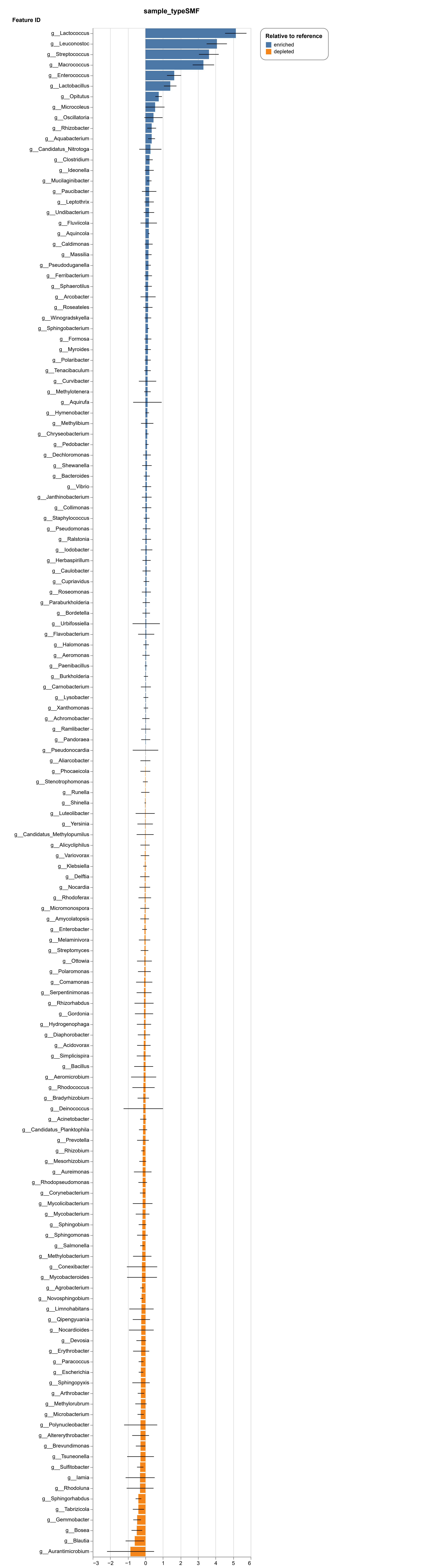

Figure S3. Differential abundance analysis in full length of the shotgun metagenomics samples from Iskar River - all DNA extraction kits were pooled in VF (reference) and SMF groups and compared. Only taxa with *p* value < 0.5 were displayed.

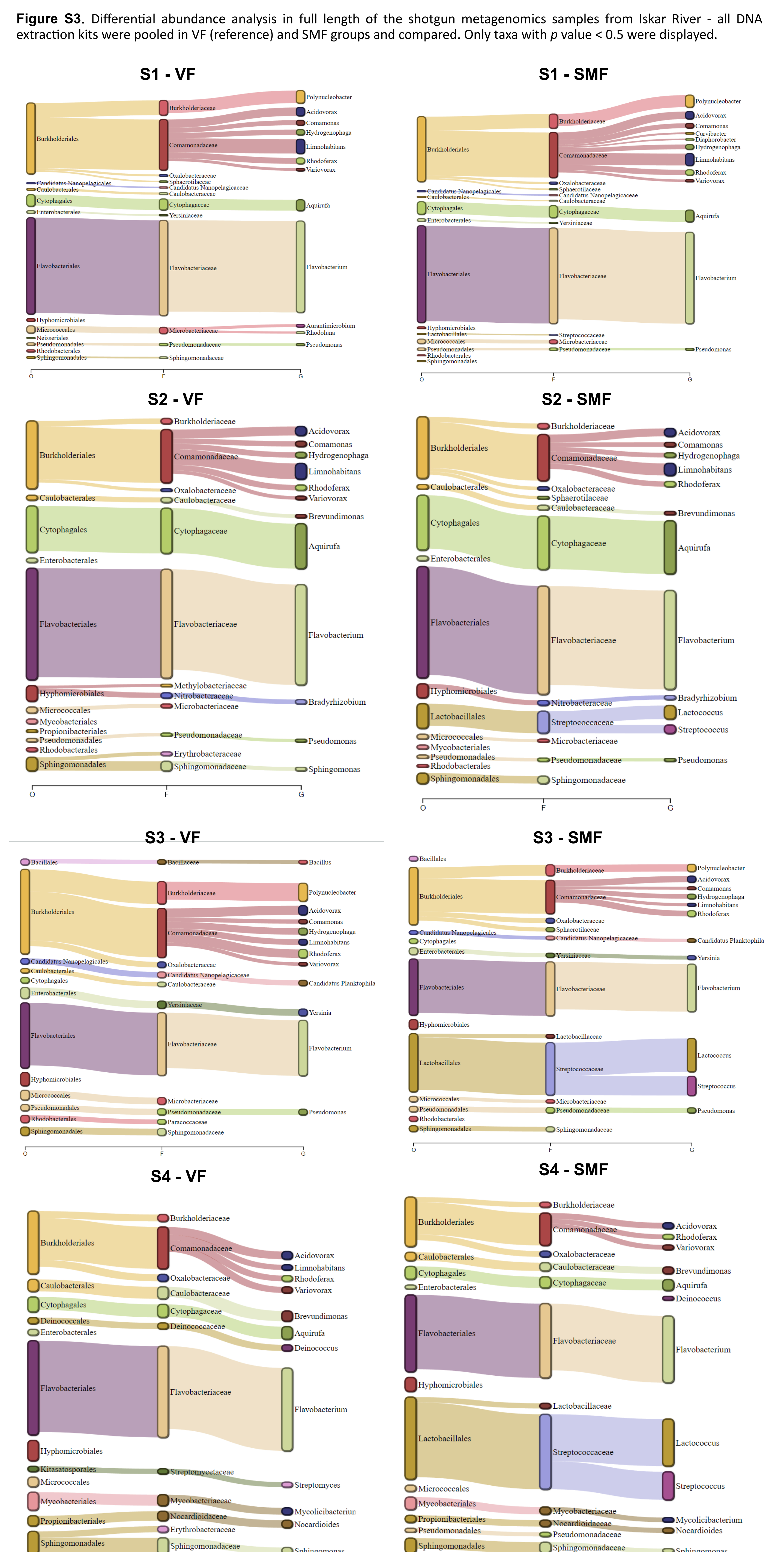

Figure S4. Sankey plots of Bracken Genus-level reports. On the left side are the samples treated with Vacuum Filtration (VF), whereas the Skimmed milk Flocculation (SMF) samples are on the right side. The plots were generated from Pavian Metagenomics Data Explorer (https://fbreitwieser.shinyapps.io/pavian/).
